# Genetic Parameter Estimation of White Spot Traits in the Carapace of Swimming Crab *Portunus trituberculatus*

**DOI:** 10.3390/ani16142123

**Published:** 2026-07-08

**Authors:** Jiahui Liu, Ouwen Shi, Shaokun Lu, Ronghua Li, Jianyu Xu, Hao Cui, Run Tong, Chunlin Wang, Changkao Mu, Weiwei Song, Ce Shi

**Affiliations:** 1 Key Laboratory of Aquacultral Biotechnology, Ningbo University, Chinese Ministry of Education, Ningbo 315211, China; 2 Collaborative Innovation Center for Zhejiang Marine High-Efficiency and Healthy Aquaculture, Ningbo 315211, China; 3 Key Laboratory of Green Mariculture (Co-Construction by Ministry and Province), Ministry of Agriculture and Rural, Ningbo 315211, China; 4 Faculty of Information Science and Technology, Ningbo University, Ningbo 315211, China

**Keywords:** *Portunus trituberculatus*, white spot traits, heritability, genetic and phenotypic correlation

## Abstract

The distribution of white spots on the shell is one of the remarkable characteristics of the appearance of *Portunus trituberculatus*, but there have been no reports on its characteristics and genetic analysis. In this study, seven parameters of white spots were extracted based on computer vision, including white spot number (WSN), white spot area (WSA), white spot regional area (WSRA), percentage of white spot area (PWSA), percentage of white spot regional area (PWSRA), white spot number of white spot regional area (WSNRA), and white spot number of carapace area (WSNCA). Using 22 full-sib families, we estimated genetic parameters of these white spot traits and their genetic correlations with growth carapace area (CA). Results demonstrated that these parameters exhibit continuous variation, but the performance of these white spot indicators was not consistent. Based on comprehensive principal component analysis (PCA), they could be categorized into three distinct groups within the full-sib families. The heritability estimated under the animal model ranged from 0.00 to 0.35 for white spot traits and was 0.18 for CA. Most white spot traits showed low to moderate heritability. The genetic correlations among WSN, WSA, WSRA, PWSA, and CA were significantly positive, indicating that selection for one of these traits would result in the improvement of others. This study presents the first estimation of characteristics and genetic parameters of white spot traits of *P. trituberculatus*, providing valuable information for understanding these traits and facilitating the optimization of breeding strategies for this species.

## Simple Summary

Swimming crab (*Portunus trituberculatus*) is an important marine economic crustacean widely distributed along the coastal regions of East and Southeast Asia, and it is also a significant aquaculture species in China. The distribution of white spots on the shell is one of the remarkable characteristics of the appearance of *P. trituberculatus*, but there have been no reports on its characterization and genetic analysis. This study extracted white spot characteristics using computer vision and estimated their genetic parameters based on an animal model. Our results showed that all white spot traits exhibit continuous variation. Certain traits exhibit moderate heritability (e.g., 0.30 for white spot number). The genetic correlation between some of the white spot traits and growth-related traits was significantly positive (e.g., the genetic correlation between the white spot area and the carapace area was 0.92), indicating that selection for one of these traits would result in the improvement of others. This study presents the first estimation of characteristics and genetic parameters of the white spot traits of *P. trituberculatus*, which also demonstrates that selection for growth-related traits can be conducted through visual assessment.

## 1. Introduction

The coloration of animal phenotypes is generally regulated by two mechanisms: pigment cells and structural coloration [1], which have direct impacts on species recognition, predation, and mate selection [2]. The formation of carapace coloration patterns in crustaceans occurs through the acquisition of pigments from food, which bind to carriers in pigment cells [3,4]. These pigment cells aggregate in the exoskeleton, creating protective coloration, primarily composed of carotenoids [5]. For ornamental aquatic species, such as *Cyprinus carpio haematopterus* [6], *Caridina spongicola* [7], and *Amblygobius phalaena* [8], coloration is a key economic trait influencing market value profoundly. In economically valuable aquatic animals, body color and patterns have certain economic value in themselves and significantly influence market prices. For instance, *Salmo salar* with pink flesh is more favored by consumers and commands a relatively higher price [9]; golden shell color of *Crassostrea gigas* is priced significantly higher than its differently colored counterparts [10]; consumers are more willing to pay higher prices for *Larimichthys crocea* with a brighter yellow abdomen [11]. Moreover, some colors are positively correlated with economically important traits such as growth and harvest weight [12,13,14], making color characteristics one of the promising traits in the genetic improvement research of aquatic animals [15].

*Portunus trituberculatus* is an important marine economic crustacean widely distributed along the coastal regions of China and is also a significant aquaculture species [16]. The carapace of *P. trituberculatus* is characterized by a dull green to brown color, with white spots distributed on the posterior margin, varying in shape, size, and number. Based on computer vision technology, a method for assessing the white spot traits of *P. trituberculatus* has been developed, facilitating the research of this trait and its relationship with other phenotypes [17].

**Figure 1:**
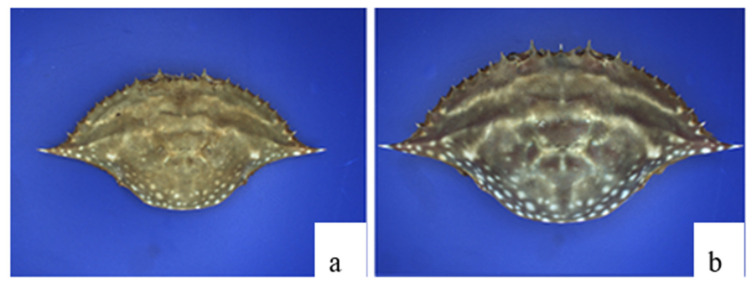
The carapace and white spots of the same *P. trituberculatus* (**a**): before molting; (**b**): after molting. From Lu et al. (2018) [17].

Growth of crustaceans is a discontinuous process. Molting (ecdysis) is a critical biological process that allows crabs to grow in size by shedding their current hard exoskeleton and producing a new one. The white spot on the carapace also changes with molting. By comparing the white spot traits of the carapace before and after molting, Lu et al. (2018) found that the number and relative position of white spots did not change after molting, whereas the white spot area and its distribution area grew with the carapace, exhibiting continuous variation (Figure 1) [17]. However, the genetic characteristics and basis of white spot traits have not been elucidated.

In this study, the characteristics and genetic parameters of the white spot traits on the carapace of *P. trituberculatus* were estimated based on full-sib families. Additionally, their relationship with growth (carapace area) was estimated to determine the feasibility of including these traits simultaneously in the breeding objective of genetic improvement programs for *P. trituberculatus*.

## 2. Materials and Methods

### 2.1. Establishment of Families and Rearing

Sexually mature and unmated *P. trituberculatus* were obtained in August from 50 independent culture ponds in Zhejiang, China, to construct sib families. The broodstock for each pond originated from 10–15 distinct wild berried females.

From each pond, six crabs (150.4 ± 12.6 g, female: male = 1:1) were selected, with priority given to individuals exhibiting greater variation in carapace white spots. During the one-week acclimatization, crabs were kept in concrete ponds (5 m length × 4 m width × 1.2 m depth) under the following conditions: water temperature 20 ± 1 °C, salinity 28 ± 1‰, pH 8.0 ± 0.2, dissolved oxygen ≥ 5.0 mg/L, and natural photoperiod (approximately 12L:12D). They were fed daily with fresh Manila clam (*Ruditapes philippinarum*) at 5% of body weight; uneaten food was removed 2 h after feeding. One-third of the water was exchanged daily. After a week of acclimatization, they were used to construct 150 full-sib families, with a single male and a female kept in a plastic basket (63 cm × 43 cm × 38 cm). All successfully mated females were moved to a greenhouse pond (2.5 acres, 2 m depth) in November for wintering. In April of the following year, broodstock crabs ready to spawn were transferred to nursery concrete ponds, with one crab placed in each pond to create full-sib families. Spawning occurred approximately 10–15 days after transfer. Prior to spawning, an appropriate quantity of rotifers was introduced into the nursery ponds. During the first zoea (Z1, 1–2 days post-hatching) and second zoea (Z2, 3–5 days post-hatching) stages, the larvae were fed a diet of rotifers and artificial compound feed (Raoping Yongdeli Feed Co., Ltd., Chaozhou, Guangdong, China) (crude protein ≥45%, crude fat ≥5%, crude fiber ≤13%, crude ash ≤16%, and moisture ≤10%). During the third zoea (Z3, 6–8 days post-hatching) and fourth zoea (Z4, 9–11 days post-hatching) stages, they were fed with brine shrimp (*Artemia*) nauplii, and during the megalopa stage (M, 12–14 days post-hatching), frozen adult brine shrimp served as the primary feed. For each full-sib family, crablets were harvested once the majority of megalopa had metamorphosed into first-stage crablets (CI, 15–20 days post-hatching), a process that took about 23 days. A total of 22 full-sib families were successfully established and transferred to separate earth ponds for the following rearing. During earth pond culture, adequate Manila clam (*Ruditapes philippinarum)* and frozen fish were provided once per day at 16.00 with a feeding amount of 10% of crabs based on total biomass. Total biomass was estimated using the mixed body weight estimation method from the growth model described by Lu et al. [18]. The next morning at 8.00 am, one-third of the water was exchanged with continuous aeration.

### 2.2. Sample Collection and Trait Measurement

**Figure 2:**
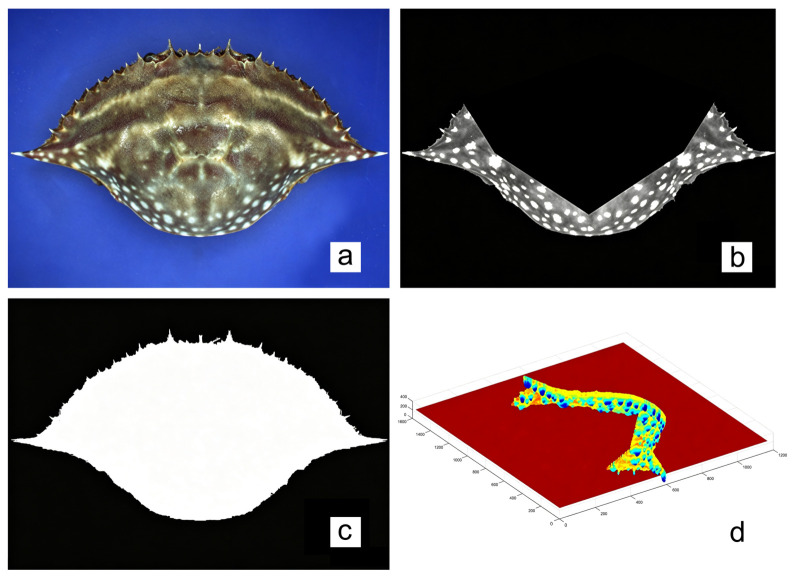
Diagram of the carapace image process: (**a**) original image; (**b**) grayscale image; (**c**) binarization; (**d**) watershed divide image. From Lu et al. (2018) [17].

**Figure 3:**
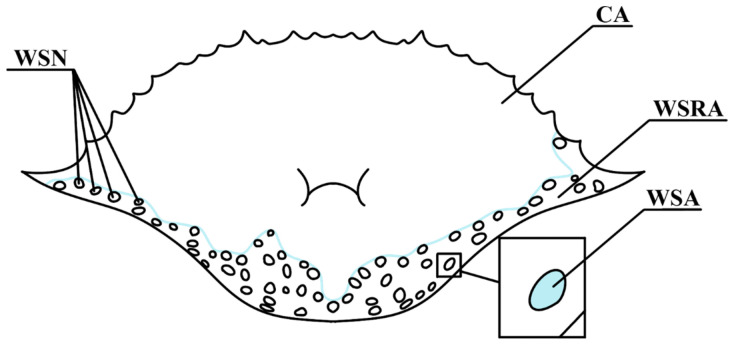
Schematic diagram of white spot traits measured in *P. trituberculatus.* Note: WSN—white spot number; WSA—white spot area; WSRA—white spot regional area; CA—carapace area. The blue spot demonstrates a representative of WSA, and the area enclosed by the blue lines represents WSRA.

After 90-day pond culture, a total of 924 crabs from 22 families were randomly selected for the measurement of carapace area, and the white spot traits on the carapace were analyzed following the computer vision methods established by Lu et al. [17] (Figure 2). In brief, the image acquisition device for carapace consists of an industrial camera (JAI CB-200GE, 5 mm lens) (JAI Inc., San Jose, CA, USA), an Ethernet cable, a computer (ThinkPad E430) (Lenovo Group Ltd., Beijing, China), a white square box (40 cm × 20 cm × 28 cm), and a fluorescent lamp (30 W white light source) (Foshan Electrical and Lighting Co., Ltd., Foshan, Guangdong, China). Both the camera and the fluorescent lamp are installed above the square box, and the camera is connected to the computer. Images of the carapaces were captured and saved in TIFF format. OpenCV (version 4.0.0) was utilized to convert the calibrated images to grayscale, followed by a binarization process. The watershed segmentation (Otsu’s Thresholding) was employed to identify the traits on the carapace, including the white spot number (WSN, the total number of white spots), white spot area (WSA, the total area of white spots), white spot regional area (WSRA, the area enclosed by a polyline connecting the white spots closest to the upper margin of the carapace and the posterior margin of the carapace), and carapace area (CA, the surface area of crab shell) as indicated in Figure 3.

To verify the accuracy of the computer recognition program, 30 crab carapace images were randomly selected. Manual delineation and computer recognition processing were performed separately, and the resulting data were compared and analyzed. The analysis results showed that for all the white spot traits and carapace area, there were no significant differences (*p* > 0.05) between the values obtained by manual delineation and computer vision recognition, with a similarity of 100% [17].

Percentage of white spot area (PWSA), percentage of white spot regional area (PWSRA), white spot number of white spot regional area (WSNRA), and white spot number of carapace area (WSNCA) were calculated according to the following formulas:

PWSA (%) = WSA/CA × 100

PWSRA (%) = WSRA/CA × 100

WSNRA = WSN/WSRA × 10,000

WSNCA = WSN/CA × 10,000

Carapace and white spot traits of parents were measured once the spawning had finished (data for the parents of two families were missing and were not included in the correlation analysis of white spot traits between parents and offspring).

### 2.3. Characteristic Analysis of White Spot Traits

Phenotypic data for carapace and white spot traits (WSN, WSA, WSRA, CA, PWSA, PWSRA, WSNRA, and WSNCA) were analyzed using SPSS 22.0 (IBM, Armonk, NY, USA). The phenotypic data for the eight traits from broodstock were tested for normal distribution using histogram and Shapiro–Wilk test [19]. The data that did not meet the normal distribution were subjected to natural logarithmic or Box–Cox transformations before calculating the genetic variance [20]. The correlation analysis (Pearson correlation analysis and Spearman correlation analysis) of these traits between parents and offspring was also performed [21]. A significance level of *p* < 0.05 was considered statistically significant, and *p* < 0.01 was considered extremely significant.

Bartlett’s test was employed to verify whether the data satisfied the prerequisites for PCA. Principal component analysis (PCA) was carried out to evaluate the patterns of trait combination and overall morphological variation [22]. All the eight traits were standardized using the following formula to transform them into a standard normal distribution with a mean of 0 and a standard deviation of 1.

$$Z=\frac{x-\mu }{\sigma }$$

x is the original data, μ is the mean of the trait, and σ is the standard deviation of the trait.

Cluster analysis was carried out to obtain a comprehensive evaluation of white spot traits in the full-sib families using Euclidean distance as a similarity measure [23].

### 2.4. Heritabilities and Correlation Analysis

Genetic parameters of white spot traits were estimated using the following model in ASReml (ASReml-R package) [24]:

$$y=Xb+Zu+Wp_{o}+e$$

where *y* represents the vector of observations, *b* is the vector of fixed effects due to overall mean, *u* is the vector of random animal effects, and *e* is the vector of random residual effects. It was assumed that random effects (*u* and *e*) are normally distributed; u ~ N (0, A $\sigma_{\alpha }^{2}$), where $\sigma_{\alpha }^{2}$ is the additive genetic variance and A is the additive genetic relationship matrix derived from the pedigree; e ~ N (0, I $\sigma_{e}^{2}$), where $\sigma_{e}^{2}$ is the residual variance, and I is the identity matrix. The *X* and *Z* are the design matrices relating observations to the levels of fixed effects and random effects, respectively.

The pond effect was treated as a random effect in this model to identify any confounding between pond effect and additive genetic effects. It was assumed that the pond effects ($p_{o}$) are random and normally distributed; $p_{o}$ ~ N (0, I $\sigma_{p_{o}}^{2}$) where $\sigma_{p_{o}}^{2}$ is the pond variance and I is the identity matrix. W is the design matrix relating observations to the levels of random effects.

Heritability (*h*^2^) was estimated using the variance components derived from the animal model:

$$h^{2}=\frac{\sigma_{\alpha }^{2}}{\sigma_{\alpha }^{2}+{\sigma_{p_{o}}^{2}+\sigma }_{e}^{2}}$$

Genetic ($r_{g}$) and phenotypic ($r_{p}$) correlations between traits were calculated using the following covariance–variance ratio:

$$r_{x,y}=\frac{\sigma_{xy}}{\sqrt{\sigma_{x}^{2}\sigma_{y}^{2}}}$$

For genetic correlations, $\sigma_{xy}$ refers to the additive genetic covariance between traits *x* and *y*, with $\sigma_{x}^{2}$ and $\sigma_{y}^{2}$ representing their respective additive genetic variances. For phenotypic correlations, the covariance and variance components correspond to the phenotypic level. Statistical significance testing was conducted using the Likelihood Ratio Test.

## 3. Results

### 3.1. Descriptive Statistics of White Spot Traits

The phenotypic parameters for the carapace and white spot traits of the broodstock are presented in Table 1. Among the eight traits examined, the coefficient of variation (CV) ranged from 27.65% (for WSNRA) to 75.14% (for WSA), indicating that the white spot traits exhibit considerable genetic variability and high selection potential.

**Figure 4:**
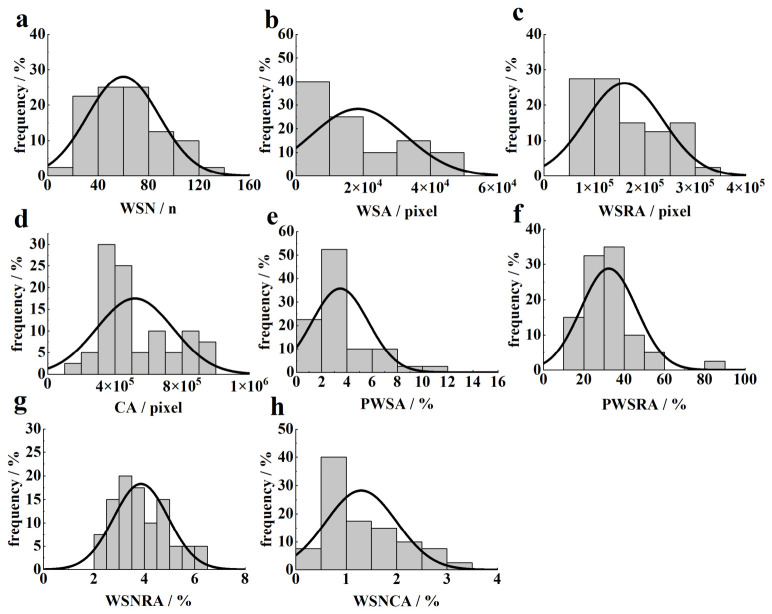
Histograms of white spot traits in the broodstock of *P. trituberculatus*. (**a**) WSN; (**b**) WSA; (**c**) WSRA; (**d**) CA; (**e**) PWSA; (**f**) PWSRA; (**g**) WSNRA; (**h**) WSNCA. Note: WSN—white spot number; WSA—white spot area; WSRA—white spot regional area; PWSA—percentage of white spot area; PWSRA—percentage of white spot regional area; WSNRA—white spot number of white spot regional area; WSNCA—white spot number of carapace area; CA—carapace area.

Among the eight appearance indicators, the WSN (*W* = 0.963, *p* > 0.05) and WSNRA (*W* = 0.952, *p* > 0.05) followed continuous normal distributions. In contrast, the other six indicators demonstrated a skewed distribution (*p* < 0.05) (Figure 4).

The correlation analysis of white spot traits between full siblings and their parents demonstrated a significantly positive correlation in the WSN and PWSA values between offspring and the female parent using Pearson and Spearman correlation analysis (Table 2).

### 3.2. Principal Component Analysis and Family Cluster of White Spot Traits

The Bartlett’s test indicated that the data were suitable for PCA (*p* < 0.001). Principal component analysis (PCA) was performed on the phenotypic traits (Table 3). The results demonstrated that the eigenvalues for the first two principal components were greater than 1, which collectively accounted for 84.96% of the total variance. The information loadings of associated factors in each principal component demonstrated that PC1 and PC2 were driven by a small number of variables with clear characteristics (Table 4). PC1 was positively correlated with WSN, PWSA, and WSA, which reflected the number and distribution area of white spots. CA and WSNRA had a larger factor loading on PC2, which reflected the density of white spots on the carapace.

**Figure 5:**
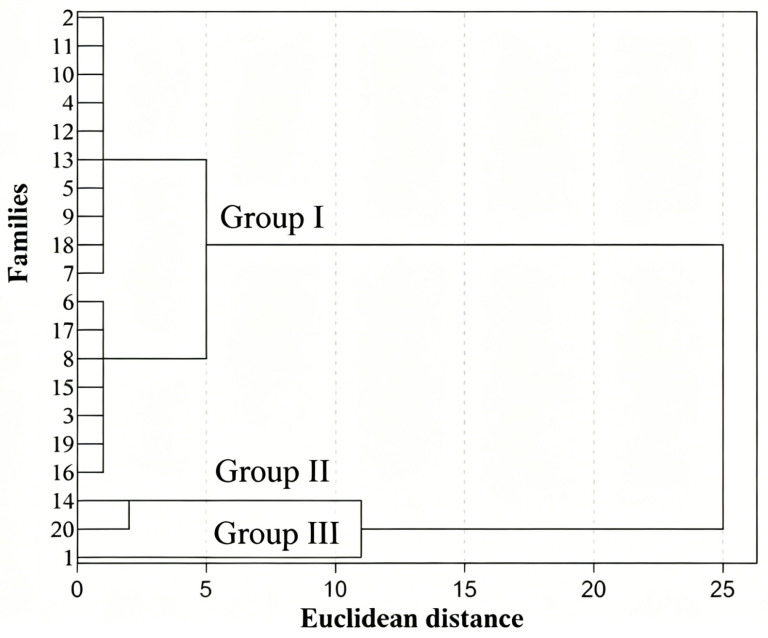
Cluster analysis of full-sibling families based on white spot traits in the carapace. Representative photographs of the three subgroups are presented in Figure S1.

The raw data of the offspring’s spot traits from different families were standardized and subjected to comprehensive scoring and ranking (Table 5). Families 13, 4, and 12 received higher scores, indicating that these were white spot families enriched with white spots from a comprehensive perspective based on PC1 and PC2. The results of the cluster analysis on all spot traits of the progeny from the full-sibling families revealed that these families could be categorized into three distinct groups with a Euclidean distance coefficient of 10 (Figure 5). Group I included 17 families characterized by a higher score in PC1 and PC2 (relatively abundant white spot number and concentrated distribution). Group II included family 14 and family 20, characterized by a medium score in PC1 and lower PC2 score (relatively abundant white spot number and scattered distribution). Group III included family 1, which was characterized by a lower score in both PC1 and PC2 (lower white spot number and scattered distribution). Representative photographs for the three subgroups are provided in Figure S1.

### 3.3. Estimation of Genetic Parameters and Correlations Analysis

Heritability estimates for all white spot-related traits ranged from 0.00–0.35 (Table 6). Most white spot traits showed moderate heritability, such as WSN (0.30 ± 0.12), WSA (0.32 ± 0.11), WSRA (0.29 ± 0.12), and PWSA (0.35 ± 0.11). The heritability for the growth-related trait of carapace area (CA) was 0.18 ± 0.12.

The phenotypic and genetic correlations among WSN, WSA, WSRA, and PWSA were high and significantly positive (0.18–0.93 for phenotypic correlation, 0.51–0.99 for genetic correlation). Besides, the phenotypic and genetic correlations between some of the white spot-related traits (WSN, WSA, WSRA, PWSA) and CA were also significant (*p* < 0.05 or *p* < 0.01 as indicated in Table 6).

## 4. Discussion

Analyzing and evaluating the morphological characteristics and genetic parameters of color phenotype traits serve as the foundation for the identification [25], conservation [26], and breeding of germplasm resources. The white spots on the shell are one of the remarkable characteristics of the appearance of *P. trituberculatus*, but there have been no reports on the genetic analysis of this characteristic. In this study, traits of white spots were identified based on computer vision, and genetic parameters of the white spot traits and their relationship with growth were primarily estimated based on full-sib families.

The biological functions of white spot traits in *P. trituberculatus* remain largely unknown. Preliminary research indicates that in crustaceans, body coloration is primarily determined by carotenoid pigments obtained from the diet and stored in chromatophores [3,4]. The number, size, and distribution of white spots could reflect differences in carotenoid deposition efficiency, chromatophore density, or structural color mechanisms [1]. The fact that white spots are located on the exoskeleton and their area expands with molting without changing their number or relative position [17] suggests they may be linked to exoskeleton mineralization or cuticle layer formation. The white spots could also have adaptive significance, since the dull green-to-brown background color of the carapace, combined with white spots, might serve as camouflage similar to sandy or rocky seabeds, reducing predation risk [1]. Future studies combining physiological and behavioral experiments are needed to deeply elucidate the biological roles and mechanism of white spots in this species.

This study demonstrates that white spot traits of *P. trituberculatus* exhibit continuous variation, which is a characteristic of quantitative traits [27]. Notably, WSN and WSNRA exhibit the typical quantitative genetic characteristic of a normal distribution. It is generally accepted that the phenotypes of quantitative traits controlled by multiple minor effect genes tend to follow a normal distribution. However, the other white spot traits display a skewed distribution. One possible explanation is that major-effect genes influence these quantitative traits, along with non-additive effects, gene linkage, or interactions, which in turn lead to the skewed distribution of phenotypes [27]. For instance, in maize (*Zea mays* L.), major quantitative trait loci (QTLs), including additive and epistatic QTLs, have been identified, which contribute directly to the phenotypic variation of husk number (HN) and husk length (HL) [28]. In Cichlidae, a hybrid cross between the horizontally striped *Pseudotropheus cyaneorhabdos* and the vertically barred *Chindongo demasoni* revealed that horizontal stripes are controlled by a few major-effect loci, whereas vertical bars are governed by many polygenic genes [29]. In *P. trituberculatus*, the genome has also been assembled, which could provide a crucial foundation for future efforts to identify the genes controlling the white spot traits [30]. It should be noted that non-genetic factors such as environmental variation, measurement artifacts, or scaling effects may also contribute to the skewed distribution of some white spot traits. For instance, researchers have confirmed that the substantial phenotypic skew in the body size of *Cyanistes caeruleus* is driven by environmental factors [31], which should be considered in future studies.

The coefficients of variation for the eight appearance traits ranged from 27.65% to 75.14%, indicating abundant genetic variability and selection potential of these traits [32]. A significantly positive correlation was observed in the WSN and PWSA values between offspring and the female parent using Pearson and Spearman analysis, suggesting that these two white spot traits may be partially maternally inherited, which will need further investigation.

In family studies, a large number of parameters related to the characteristics of target traits are often collected from family members. Principal component analysis is a useful tool for the comprehensive evaluation of these parameters [33]. Principal component analysis of the white spot traits in *P. trituberculatus* indicated that variation in the various white spot indices was evident. Based on PC1 and PC2, families could be categorized into three distinct groups characterized by (1) relatively abundant white spot number with concentrated distribution; (2) relatively abundant white spot number with scattered distribution; (3) lower white spot number with scattered distribution. These results demonstrated that the performance of various white spot parameters in the carapace is not consistent, and independent analysis is necessary for the understanding of each white spot trait.

Based on the phenotypic differentiation observed among families, we proceeded to estimate the heritability of white spot traits. Estimation of genetic parameters is the foundation for carrying out a selective breeding program [27]. Studies on shell color in *C. gigas* have shown that the heritability of black, golden, and purple shell colors all falls within the moderate-to-high range [34,35]. Similarly, the heritability of spot intensity below the lateral band (SB) in rainbow trout (*Oncorhynchus mykiss*) was estimated as 0.44 ± 0.04 [14], indicating that certain color traits can be improved through selective breeding programs. This study presented the first report of the heritability of white spot traits in the carapace of *P. trituberculatus*. In this preliminary study, we found that most white spot traits showed moderate heritability, such as WSN (0.30 ± 0.12), WSA (0.32 ± 0.11), WSRA (0.29 ± 0.12), and PWSA (0.35 ± 0.11), reflecting the joint influence of genetic and environmental components. Family selection or genomic approaches can be employed for selective breeding of these traits. The heritability of growth-related traits in crustaceans generally falls within a medium-to-high range. For instance, in *Penaeus vannamei*, the heritability of body weight at 150 and 240 days of age was 0.52 ± 0.09 and 0.44 ± 0.07, respectively [36]. Hasan et al. (2022) reported that the heritabilities of body length, body weight, body shape, head size, abdominal size, and telson tip in *Penaeus monodon* were 0.36 ± 0.06, 0.32 ± 0.05, 0.32 ± 0.05, 0.31 ± 0.05, 0.32 ± 0.05, and 0.28 ± 0.05, respectively [37]. In the present study, the estimated heritability of carapace area (CA) was 0.18 ± 0.12. This indicates that carapace area, as a growth indicator, does not exhibit heritability as high as other metrics such as body weight and body length.

In aquaculture breeding schemes, growth is a relatively important trait of concern. Several morphological traits were found to be significantly genetically correlated with growth, e.g., the sixth segment width of *P. vannamei* [38] and the black shell color of *C. gigas* [13] were genetically correlated with weight, which could be improved simultaneously through selective breeding programs. In this study, significant genetic correlations were detected among some of the white spot traits (WSN, WSA, WSRA, PWSA) and CA, suggesting that selecting for these white spot traits can lead to positive selection for CA or vice versa. However, given the mathematical relationships among some of these variables (e.g., PWSA = WSA/CA), this high correlation may be partly driven by the derived nature of the variables rather than true genetic pleiotropy and should be interpreted with caution.

The present study provided a preliminary estimate of heritability for the white spot traits using full-sib families in a separately reared condition; however, this design itself has inherent limitations: it does not allow accurate separation of additive genetic effects from common environmental, dominance, maternal effects, and the pond and family effects cannot be disentangled when each family is reared in a single pond, which may result in an upward bias in heritability estimates. Previous research has shown that common environmental effects arising from physical separation of full-sib families can account for 5–55% of the phenotypic variation in economic traits in aquaculture species. Ignoring these effects in genetic evaluation can lead to a 9–45% overestimation of heritability and may alter the ranking of selection candidates [39]. Future studies should adopt more rigorous designs, such as mixed rearing in the same pond with molecular parentage identification and constructing half-sib families, to obtain more accurate estimates of genetic parameters.

## 5. Conclusions

This study presents the first estimation of characteristics and genetic parameters of the white spot traits on the carapace of *P. trituberculatus* based on full-sib families. A total of seven parameters were used to depict the traits of white spots. Results demonstrated that these parameters exhibit the characteristic of continuous variation, but the performance of these white spot parameters in the carapace was not consistent, and they could be categorized into three distinct subgroups according to the PCA analysis. The preliminary estimated heritability for most of the white spot traits was at a medium level, indicating that they were influenced by genetic and environmental factors and have potential for genetic improvement. The genetic correlation among some of the traits was high and significantly positive, indicating that selection for one of these white spot traits would result in the improvement of others. In the present study, the full-sib family design may result in an upward bias in heritability estimates; future studies should adopt more rigorous designs, such as mixed rearing in the same pond and constructing half-sib families, to obtain more accurate estimates of genetic parameters.

## Figures and Tables

**Table 1:** Parameters of white spot traits in the broodstock of *P. trituberculatus*.

Index	Maximum	Minimum	Standard Error	Mean	Coefficient of Variation (%)
WSN	120	13	28.07	60.08	46.72
WSA (pixel)	4.91 × 10^4^	2.63 × 10^3^	1.39 × 10^4^	1.85 × 10^4^	75.14
WSRA (pixel)	3.26 × 10^5^	5.92 × 10^4^	7.53 × 10^4^	1.59 × 10^5^	47.36
CA (pixel)	9.94 × 10^5^	1.83 × 10^5^	2.28 × 10^5^	5.19 × 10^5^	43.89
PWSA (%)	10.43	0.70	2.21	3.47	63.69
PWSRA (%)	55.01	10.24	10.25	31.17	32.88
WSNRA (%)	6.23	2.19	1.07	3.87	27.65
WSNCA (%)	3.06	0.22	0.71	1.30	54.38

**Table 2:** Correlation analysis of the white spot traits between full siblings and their parents.

Traits	Bivariate Parameter	Pearson Correlation	*p*	Spearman Correlation	*p*
WSN	progenies&parents	0.466	0.038 *	0.399	0.082
	progenies&dam	0.467	0.038 *	0.511	0.021 *
	progenies&sire	0.387	0.092	0.303	0.194
WSA	progenies&parents	0.148	0.535	0.120	0.613
	progenies&dam	0.194	0.412	0.144	0.544
	progenies&sire	0.031	0.897	0.080	0.975
WSRA	progenies&parents	0.123	0.606	−0.029	0.905
	progenies&dam	0.168	0.478	0.033	0.890
	progenies&sire	0.006	0.981	−0.032	0.895
CA	progenies&parents	−0.011	0.963	−0.313	0.179
	progenies&dam	0.040	0.987	−0.302	0.195
	progenies&sire	−0.074	0.756	−0.164	0.490
PWSA	progenies&parents	0.475	0.034 *	0.546	0.013 *
	progenies&dam	0.518	0.019 *	0.561	0.010 *
	progenies&sire	0.299	0.200	0.383	0.095
PWSRA	progenies&parents	0.223	0.344	0.415	0.069
	progenies&dam	0.070	0.769	0.274	0.243
	progenies&sire	0.409	0.074	0.490	0.028 *
WSNRA	progenies&parents	0.260	0.269	0.277	0.237
	progenies&dam	0.036	0.880	0.057	0.811
	progenies&sire	0.332	0.152	0.322	0.166
WSNCA	progenies&parents	0.273	0.245	0.387	0.092
	progenies&dam	0.081	0.734	0.172	0.468
	progenies&sire	0.369	0.109	0.372	0.107

Note: * significant difference (*p* < 0.05).

**Table 3:** The eigenvalues and cumulative variance contribution rates of evaluation factors for white spot traits in *P. trituberculatus*.

Components	Eigenvalue	Variance Contribution Rate/%	Accumulated Variance Contribution Rate/%
1	4.023	50.286	50.286
2	2.774	34.674	84.960
3	0.708	8.849	93.809
4	0.410	5.123	98.932
5	0.040	0.498	99.430
6	0.030	0.379	99.808
7	0.010	0.131	99.939
8	0.005	0.061	100.000

**Table 4:** Principal component load matrix for white spot traits in *P. trituberculatus*.

Traits	Principal Component	
	1	2
WSN	0.944	−0.030
WSA	0.812	−0.505
WSRA	0.780	−0.565
CA	0.274	−0.928
PWSA	0.887	0.086
PWSRA	0.648	0.443
WSNRA	0.363	0.780
WSNCA	0.670	0.725

**Table 5:** Principal component scores for white spot traits in full-sibling families of the *P. trituberculatus*.

Family ID	1	2	Component Score	Sort
13	−0.715	2.932	1.193	1
4	1.472	2.727	1.122	2
12	−0.582	2.621	1.066	3
10	0.384	1.920	0.786	4
2	−0.956	1.678	0.679	5
18	2.188	1.259	0.527	6
11	−1.345	1.106	0.443	7
7	0.784	0.871	0.360	8
5	−2.145	0.558	0.215	9
9	−1.233	0.438	0.171	10
17	−0.287	−0.049	−0.022	11
3	1.804	−0.304	−0.113	12
15	−0.486	−0.470	−0.195	13
6	−1.863	−0.578	−0.247	14
19	2.519	−0.869	−0.339	15
8	−1.841	−1.046	−0.438	16
16	2.968	−1.249	−0.492	17
14	−1.072	−3.247	−1.331	18
20	0.876	−3.380	−1.374	19
1	−0.472	−4.917	−2.010	20

**Table 6:** Heritability (in bold, positioned along the diagonal), genetic correlation (below the diagonal), and phenotypic correlation (above the diagonal) of the white spot traits in *P. trituberculatus*.

Traits	WSN	WSA	WSRA	CA	PWSA	PWSRA	WSNRA	WSNCA
WSN	**0.30 ± 0.12**	0.69 ± 0.05 **	0.65 ± 0.06 **	0.29 ± 0.09 **	0.79 ± 0.03 **	0.53 ± 0.08 **	0.19 ± 0.09 **	0.35 ± 0.08 **
WSA	0.92 ± 0.11 **	**0.32 ± 0.11**	0.93 ± 0.01 **	0.80 ± 0.04 **	0.74 ± 0.05 **	0.09 ± 0.11 **	−0.50 ± 0.073 **	−0.34 ± 0.09 **
WSRA	0.71 ± 0.17 *	0.91 ± 0.05 **	**0.29 ± 0.12**	0.81 ± 0.04 **	0.54 ± 0.07 **	0.13 ± 0.11 **	−0.63 ± 0.06 **	−0.41 ± 0.09 **
CA	0.69 ± 0.32 *	0.81 ± 0.13 *	0.99 ± 0.22 *	**0.18 ± 0.12**	0.18 ± 0.10 *	−0.44 ± 0.09 **	−0.82 ± 0.03 **	−0.80 ± 0.03 **
PWSA	0.91 ± 0.09 **	0.83 ± 0.11 **	0.51 ± 0.20 *	/	**0.35 ± 0.11**	0.57 ± 0.08 **	0.09 ± 0.10 **	0.33 ± 0.09 **
PWSRA	/	/	/	/	/	**0.00 ± 0.06**	0.39 ± 0.09 **	0.72 ± 0.05 **
WSNRA	/	/	/	/	/	/	**0.25 ± 0.12**	0.92 ± 0.02 **
WSNCA	/	/	/	/	/	/	/	**0.09 ± 0.11**

Note: data are presented as mean ± SE, / indicates no value or not significant, * indicates significantly correlated (*p* < 0.05), ** indicates extremely significantly correlated (*p* < 0.01).

## Data Availability

The original contributions presented in this study are included in the article and Supplementary Material. Further inquiries can be directed to the corresponding author.

## Supplementary Materials

The following supporting information can be downloaded at: https://www.mdpi.com/article/10.3390/ani16142123/s1, Figure S1: Representative photographs of the carapace of the three subgroups showing white spot patterns.
