# Supplementary material for: Genetic Parameter Estimation of White Spot Traits in the Carapace of Swimming Crab Portunus trituberculatus
Source: Animals (Basel). 2026 Jul 8;16(14):2123. doi: 10.3390/ani16142123 (PMC13404941; doi:10.3390/ani16142123)
Supplement: Supplementary file 1 [file animals-16-02123-s001.zip › animals-4330620-supplementary.pdf]

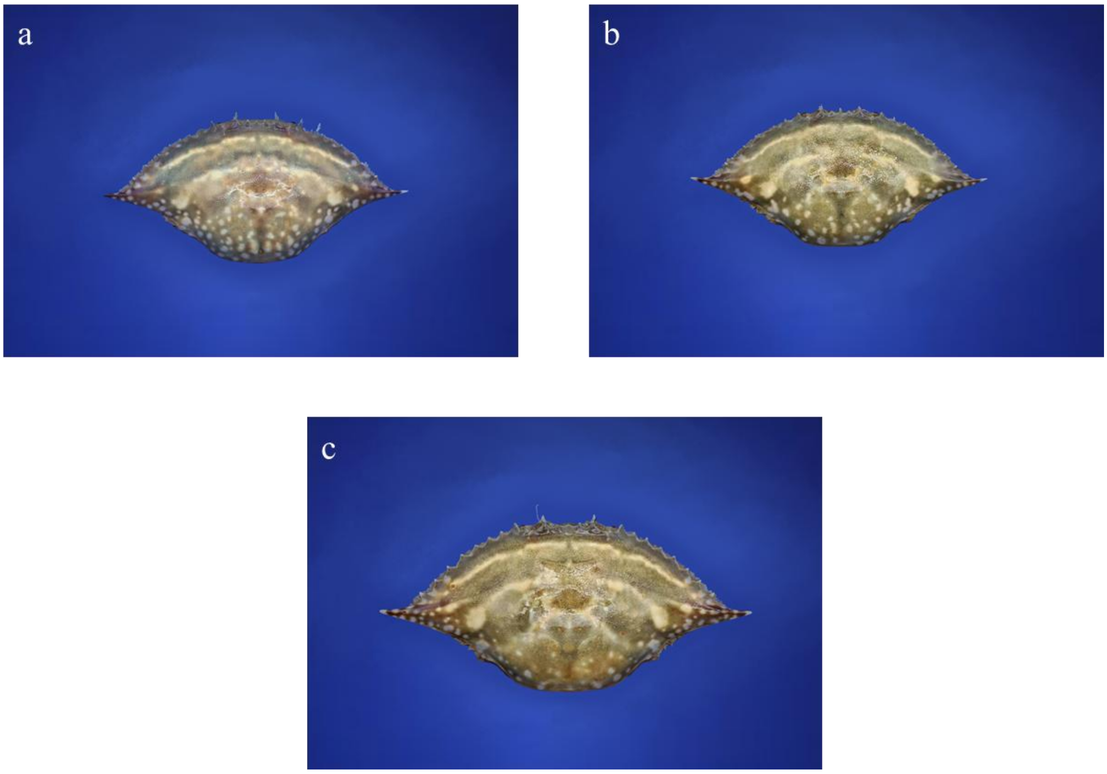

Figure S1. Representative photographs of the carapace of the three subgroups showing white spot patterns.

Note: a: Group I; b: Group II; c: Group III. Group I was characterized by relatively abundant white spot number and concentrated distribution. Group II was characterized by relatively abundant white spot number and scattered distribution. Group III was characterized by fewer white spot number and scattered distribution.
